# Three-dimensional modeling and automatic analysis of the human nasal cavity and paranasal sinuses using the computational fluid dynamics method

**DOI:** 10.1007/s00405-020-06428-3

**Published:** 2020-10-17

**Authors:** Dmitry Tretiakow, Krzysztof Tesch, Jarosław Meyer-Szary, Karolina Markiet, Andrzej Skorek

**Affiliations:** 1 Department of Otolaryngology, Gdansk Medical University, Smoluchowskiego Str. 17, 80-214 Gdansk, Poland; 2 Faculty of Mechanical Engineering, Gdansk University of Technology, Gdansk, Poland; 3 Department of Paediatric Cardiology and Congenital Heart Defects, Gdansk Medical University, Gdansk, Poland; 4 II Department of Radiology, Gdansk Medical University, Gdansk, Poland

**Keywords:** Nasal obstruction, Nasal resistance, 3D-model, Airflow, Airway, Adults

## Abstract

**Purpose:**

The goal of this study was to develop a complete workflow allowing for conducting computational fluid dynamics (CFD) simulation of airflow through the upper airways based on computed tomography (CT) and cone-beam computed tomography (CBCT) studies of individual adult patients.

**Methods:**

This study is based on CT images of 16 patients. Image processing and model generation of the human nasal cavity and paranasal sinuses were performed using open-source and freeware software. 3-D Slicer was used primarily for segmentation and new surface model generation. Further processing was done using Autodesk^®^ Meshmixer TM. The governing equations are discretized by means of the finite volume method. Subsequently, the corresponding algebraic equation systems were solved by OpenFOAM software.

**Results:**

We described the protocol for the preparation of a 3-D model of the nasal cavity and paranasal sinuses and highlighted several problems that the future researcher may encounter. The CFD results were presented based on examples of 3-D models of the patient 1 (norm) and patient 2 (pathological changes).

**Conclusion:**

The short training time for new user without a prior experience in image segmentation and 3-D mesh editing is an important advantage of this type of research. Both CBCT and CT are useful for model building. However, CBCT may have limitations. The *Q* criterion in CFD illustrates the considerable complication of the nasal flow and allows for direct evaluation and quantitative comparison of various flows and can be used for the assessment of nasal airflow.

## Introduction

Breathing is an indispensable condition for human life. Air quality, airway patency, and condition determine our quality of human life and predisposition to respiratory diseases. The nasal cavity is the segment of the respiratory tract that first encounters the inhaled air. The functioning of the entire respiratory tract depends on the quality of inhaled air leaving the nasal cavity because it is there that air is heated, humidified and cleaned. In addition, the analysis of olfactory stimuli also takes place in the nasal cavity. Various pathological changes and anatomical variations can directly or indirectly disrupt the airflow through the nasal cavity and alter the above-mentioned nasal functions [1–4].

Interestingly, it is difficult to determine what is clinically the normal anatomy of the nasal cavity? It is common clinical practice to observe normal nasal cavity in visual examination and yet the patient reports an abnormality with nasal breathing. Opposite situations also happen, when the patient does not report any problems with nasal patency despite having significant deformations of the nasal septum (e.g., nasal turbinate hyperplasia) [3, 4].

In addition to anterior and posterior rhinoscopy, the nasal cavity can be assessed using airway endoscopy, computed tomography (CT), MRI, rhinomanometry, olfactometry as well as questionnaires dedicated to the assessment of the nasal breathing quality and current ailments (a visual analog scale (VAS), WHOQOL-BREF questionnaire, SNOT-16 and its revisions with 20, 22 or 25 questions, ENS6Q, etc.) [5–7].

The development of medical imaging techniques allows acquisition of high-resolution three-dimensional (3-D) imagery using computed tomography (CT) [8, 9]. Depending on the subject matter, the tissue resolution can be excellent in unenhanced studies like in the case of bones or poor quality in the case of various soft tissues or vessels, but contrast enhancement techniques can mitigate this. Also, the air-tissue interface is well-defined in this modality owing to the high value of CT in otorhinolaryngology [10, 11].

The parallel development of high-resolution three-dimensional (3-D) image processing techniques imagery using CT has led to medical 3-D printing and in silico simulations [8, 9, 12, 13]. Although both of these relatively new techniques have shown its usefulness in numerous fields of medicine, they are still waiting for its broad clinical application [14].

Seeking better understanding of the anatomical–functional relations, we focused on Computational Fluid Dynamics (CFD) studies which involve numerical methods to solve fluid flow issues [15–20]. Due to the discretization and numerical solution of partial differential equations describing the flow, it is possible to approximate the distribution of velocity, pressure, temperature, and other parameters in the flow. Modern CFD programs allow for solving flows taking into account viscosity and compressibility, multiphase flows, flows in which chemical reactions occur, and Newtonian and non-Newtonian fluids. Current CFD simulation codes solve equations governing fluid flows (typical conservation of mass, momentum, and energy) utilizing the Finite Volume Method.

The goal of this study was to develop a complete protocol for conducting computational fluid dynamics simulation of airflow through the upper airways of adults based on their CT images. This task was split into three subgoals. First, generating a 3-D surface model of the upper airways by processing the raw, unenhanced CT scans of the head. The surface model had to be accurate from a medical standpoint and valid for further processing. Second, transforming the surface model into a high fidelity Cartesian computational mesh and finally conducting CFD simulation of airflow on the resulting mesh.

## Materials and methods

### Computed tomography scanning

Computed tomography images were obtained using GE LightSpeed VCT and Siemens Somatom Definition Flash scanners in axial planes with multiplanar reconstructions with a slice thickness of 0.6–0.75 mm, resolution of 512 × 512 pixels and pixel size of 0.3906 × 0.3906 mm. Included CT examinations were scanned with kV values of 100–120 and appropriate mAs (range 76–92). Cone-beam computed tomography (CBCT) images were obtained with a Kodak 9300 scanner with a resolution of 667 × 667 pixels, pixel size 0.250 × 0.250 mm and slice thickness of 0.25 mm.

### Image processing workflow

Two authors (a surgeon familiar with nasal CT scans and anatomy (DT) and a physician with over 3 years of expertise in image segmentation and 3-D medical printing (JMS) closely cooperated to develop and optimize the segmentation process. Image processing and modeling of the human nasal cavity and paranasal sinuses were performed using open-source and freeware software. 3D Slicer (version 4.10.2) was used primarily for segmentation and new surface model generation. Further processing was done using Autodesk^®^ Meshmixer TM (version 3.5.474).

### Segmentation

**Fig. 1:**
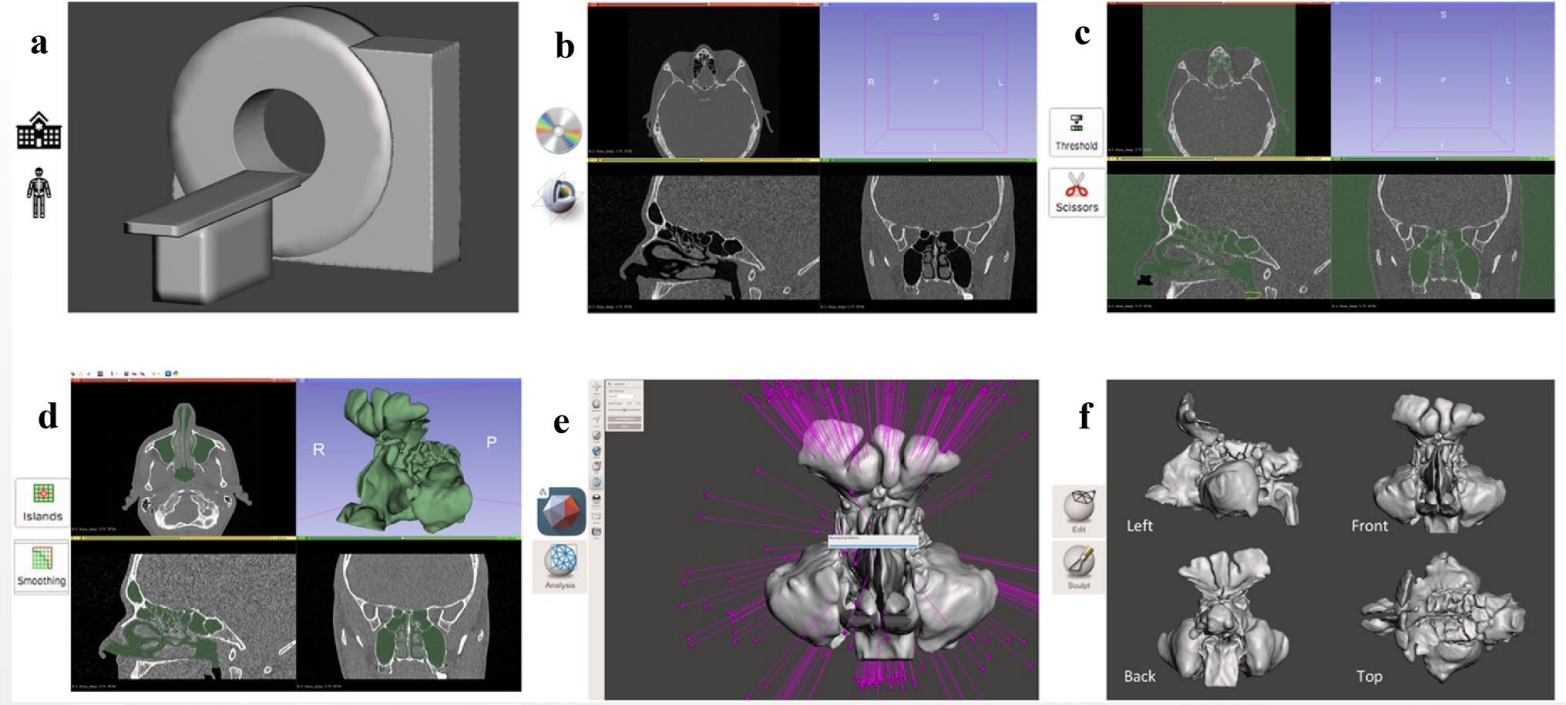
The workflow in the clinical site. **a** CT scanning, **b** loading images to 3D Slicer, image quality inspection, **c** Thresholding, and Scissors, **d** Islands and Smoothing, **e** Exporting OBJ (or STL) files and importing to Autodesk^®^ Meshmixer TM for mesh repair—Analysis-Inspector, **f** Regional editing and sculpting to achieve a final surface mash

The following workflow was developed after image acquisition (Fig. 1a). The images were read into a free, open-source software application, the 3D Slicer, as a 3-D volumetric image stack. All orthogonal views (axial, coronal, and sagittal planes) generated by the software were used for navigation and inspection of the segmentation process (Fig. 1b).

From the image segmentation toolbox, the following tools and steps were applied:

1. “threshold”: low at the minimum study level (usually − 1024 HU, Hounsfield units) and higher dependently on the image parameters, usually − 600 to − 300 HU, with careful inspection of the results by the operator (Fig. 1c),
2. “scissors” (erase inside, free-form) to separate the area of upper airways from the surroundings at the level of front nostrils and trachea at the level of nasopharynx. To succeed in the next step, it was crucial to completely isolate the nasal cavity from the surroundings in all the layers (Fig. 1c),
3. “islands” (keep selected) to isolate the target segment representing the nasal cavity and paranasal sinuses (Fig. 1d),
4. “level tracing” was used to fill holes in a mask,
5. “smoothing” (mode: median, size: one above the minimum), used optionally (if Level tracing was inadequate) to remove artifacts e.g., holes inside the mask or spikes on the edges (Fig. 1d). This step required meticulous inspection for new artifacts, especially fusion artifacts, i.e., false connecting of the neighboring but unconnected areas.

In a case of noisy images, a different approach was necessary: applying a smoothing filter onto the raw image just after reading it and before “threshold” To prevent excessive image distortion, we used Filtering, Gaussian filer with minimum useful settings.

Once the interface between the nasal mucosa and air in the nasal cavity was delineated, a 3-D surface encompassing the nasal airway was generated and was ready for output into an OBJ file format (similar to STL yet able to retain model size and unit information).

### Surface mesh inspection and fixing

Secondly, Autodesk^®^ Meshmixer TM was used to process the model further. The model was first checked for integrity, watertightness and manifold using Analysis–Inspector–Auto Repair All (Fig. 1e). Next, it was checked for any visible imperfections like holes, spikes, and unwanted connections between parts. These artifacts were manually repaired using selective editing (Select–Discard–Select–Erase and Fill) and sculpting tools (Sculpt–Brushes–Robust Smooth). These steps were crucial to the entire process and required familiarity with regional anatomy, therefore they were done by a clinician. Additionally, the model inlet and outlet were cut straight (Edit–Plane Cut) if they were found to be uneven. Finally, the model was re-meshed and reduced to 300,000–2,000,000 triangles and repaired again using the Analysis–Inspector tool. After careful inspection of the final images (Fig. 1f), the model was exported to an OBJ file.

### Computational fluid dynamics (CFD)

#### Governing equations

Since typical airflow in the nasal cavity reaches speeds < 12 m per second, and this can be regarded as incompressible flows. In this case, the air movement was described by the Navier–Stokes equation and the continuity equation. These equations describe both laminar and turbulent flow. However, due to the computational complexity in the case of turbulent flow, its average form, i.e., the Reynolds equation, is solved instead of the Navier–Stokes equation. This approach is called Raynolds-Averaged Simulation (RAS) and is the simplest way to model turbulent flow [18]. For two-equation turbulence models, two additional equations should be added. For the k-ω SST model, they are the turbulence kinetic energy transport k equation and the turbulent frequency ω transport equation [19].

#### Equation discretization

The governing equations are discretized by means of the finite volume method. Subsequently, the corresponding algebraic equation systems were solved by OpenFOAM software [20].

Divergence schemes that appear in the governing equations (convection and diffusive terms) involve Gauss integration. The discretized convection term is interpolated by means of cell-centered values. Second-order accurate linear upwind interpolation was used. Next, the discretized diffusive terms involve surface normal gradients evaluated at a cell face that connects two cells. To maintain second-order accuracy for non-orthogonal meshes, apart from orthogonal schemes, an explicit non-orthogonal and limited correction was considered.

The time derivative is discretized by means of the so-called backward differencing, meaning that this method requires the values of the unknown function at three different time steps. Additionally, this method is known to be second-order accurate in time. The transient system of equation is solved by means of the Pressure-Implicit with Splitting of Operators (PISO) algorithm [21]. The corrected pressure equation is solved by means of the Geometric agglomerated Algebraic MultiGrid preconditioner (GAMG) solver with the Diagonal Incomplete Cholesky (DIC)/Gauss–Seidel smoother. For the velocity fields and turbulent quantities standard, iterative solvers using a Gauss–Seidel smoother are utilized. Under-relaxation factors are also used to improve the stability of a solution. The assumed factors are 0.7 for velocity and 0.5 for the turbulent quantities k and ω.

#### Space and temporal discretization

The Cartesian computational mesh consists of mostly hexahedral elements. Thin layers of mesh elements around the walls were generated. This ensures that that flow near the walls was adequately resolved.

The period of the full breathing cycle (assumed to last 4 s) was divided into 4000 fixed time steps, which corresponded to 0.001 s per step. The average Courant number did not exceed 3. Moreover, the individual computing time for calculating patient 1 and patient 2 was 8.8/23.1 (laminar/turbulent) and 14.1/16.8 (laminar/turbulent) hours, respectively on a Xeon 5120 2.2 GHz processor (13 out of 14 cores involved).

#### Boundary conditions

The boundary conditions, i.e. the set of additional constraints accompanying the governing differential equations, include:

- Inlet. The volumetric flow rate (*V*) is specified according to $V = A{ \sin }\frac{2\pi t}{T}$ where (*A*) is peak amplitude, (*T*) is the period, and (*t*) is time. The typical breath period is 4 s. To obtain a volumetric flow rate of 5.1 L per minute, peak amplitude should be 16 L per minute. The specified volumetric flow rate also means that a uniform velocity field normal to the patch adjusted was forced to match the specified flow rate. Furthermore, the pressure gradient was set such that the velocity boundary condition specifies the flux on the boundary.
- Outlet. The constant total pressure distribution equal to atmospheric pressure was assumed here. It means that the outlet pressure was described by subtracting the dynamic pressure from the total pressure. Also, the velocity inlet/outlet boundary condition was specified. More precisely, a zero-gradient condition was applied for outflow, or the velocity is obtained from the patch-face normal component for inflows.
- Walls. The no-slip condition is assumed on the walls. This means that impermeability and adhesion requirements were forced. Also, the no-slip condition was accompanied by zero gradient pressure.
- The flow in the region of the near the wall in the case of turbulent flow was modeled by means of the scalable wall function. OpenFoam implementation of k-ɷ family models (SST among them) in the near wall region allows for scalable wall function if 1 < *y*^+^ < 300 or no wall function if *y*^+^ < 6. Two options are then possible, and two were inspected, giving negligible differences in terms of pressure drops. However, the former appears to be more stable.

### Statistics

Data was collected and analyzed using MS Excel 16.40. Scanning settings and image parameters were extracted from DICOM metadata. Image processing times were noted by the clinicians at 0.5 h precision. Triangles and faces count were noted for the final surface mesh, as given by Autodesk^®^ Meshmixer TM.

For continuous data, normal distribution was assumed, and the results were given as mean ± standard deviation (SD). Categorical data were presented as count and percentages.

## Results

### Patients and scanning characteristics

This study is based on medical data of 16 patients. Six healthy subjects, 1 subject with nasal septal deviation, and 5 subjects with concha bullosa and nasal septal deviation underwent CT scans for CFD modeling. The group consisted of 10 (62.5%) males and 6 (37.5%) females, ranging 27–48 years of age. All of the participants underwent medical history screening to exclude preexisting nasal sinus disease, prior nasal sinus complaints, head trauma, and prior nasal surgery. All subjects objectively confirmed the absence of severe nasal obstruction. Typically, 298–445 slices (layers) were acquired per patient. The average CTDIvol (computed tomography dose index) was 7.12 mGy and 7.42 mGy for GE and Siemens scanners, respectively. The mean effective dose for all acquisitions was approximately 0.25 mSv. One patient (#10) had a cone-beam computed tomography (CBCT) scan that features even lower radiation dose and superior spatial resolution but inferior tissue resolution (the contrast between tissues). Detailed patient and CT scanning characteristics were given in Table 1.

**Table 1:** Patient and scanning characteristics

No.	Sex	Age	Weight (kg)	Height (cm)	Modality	Resolution	Layers (n)	Slice thickness (mm)	Condition
1	M	35	79	172	CT	512 × 512	298	0.75	Normal
2	F	42	63	169	CT	512 × 512	298	0.75	DSN, CB
3	M	32	76	167	CBCT	667 × 667	428	0.25	DSN
4	M	37	81	173	CBCT	667 × 667	428	0.25	Normal
5	M	27	90	182	CT	557 × 557	445	0.75	DSN
6	M	44	97	177	CT	512 × 512	431	0.75	DSN, CB
7	F	29	59	162	CT	512 × 512	298	0.75	DSN, CB
8	M	31	87	174	CT	512 × 512	298	0.60	Normal
9	M	45	90	176	CBCT	667 × 667	428	0.25	CB
10	F	37	65	171	CBCT	667 × 667	428	0.25	Normal
11	M	42	101	185	CT	512 × 512	298	0.625	Normal
12	F	48	57	168	CT	512 × 512	298	0.625	DSN, CB
13	M	34	83	175	CT	512 × 512	368	0.75	DSN, CB
14	M	33	89	182	CT	512 × 512	298	0.625	Normal
15	F	45	71	172	CT	512 × 512	298	0.625	Normal
16	F	41	61	167	CBCT	667 × 667	428	0.25	Normal
Stat.	M 62.5%	37.2 ± 6.39	78.2 ± 14	173 ± 6.19	n/a	n/a	360 ± 66.43	n/a	n/a

*DSN* deviation septi nasi, *CB* concha bullosa, *stat*. statistical summary

### Image processing analysis

**Fig. 2:**
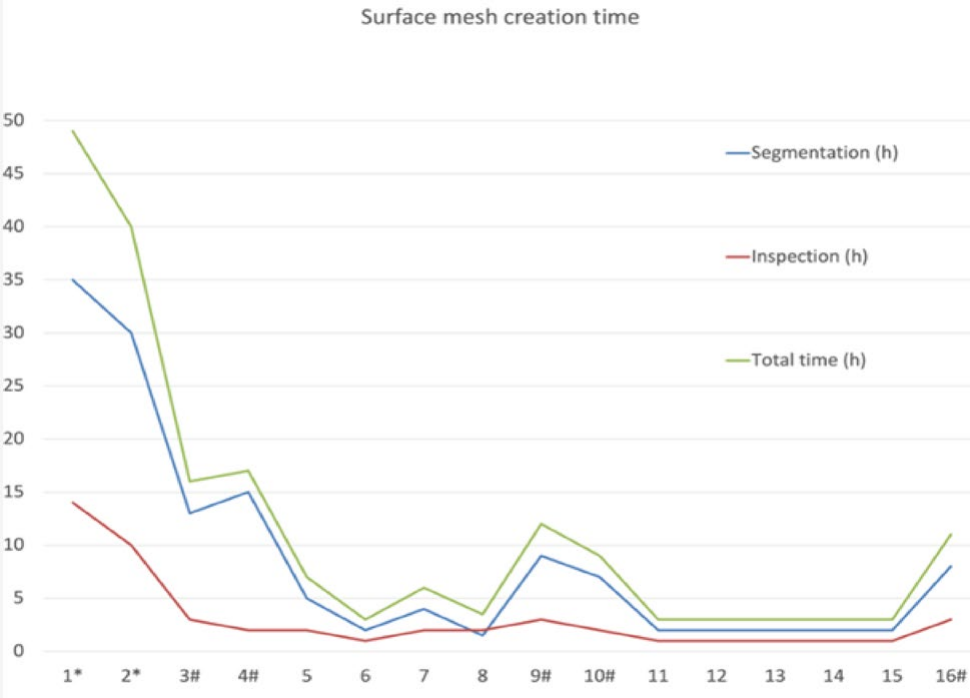
Surface mesh creation time. Segmentation was done in (3D Slicer), Mesh inspection was done in Autodesk^®^ Meshmixer TM, times are estimates with 0.5 h precision; *h* hour

The images of the first two patients were used to develop the above-described methodology and were not included in the time/workflow analysis. It took, on average, 2.95 ± 1.80 h to complete the segmentation process and 1.40 ± 0.52 h to repair and complete the surface mesh. Patient #10 took substantially longer than average due to low image quality (high noise, low tissue resolution). This scanning was done using CBCT featuring lower dose but reduced resolution and lower signal to noise ratio. CBCT image processing was attempted in four other patients (#3, 4, 9, and 16). However, it was not possible to achieve a mesh of sufficient quality that would allow successful discretization. Detailed image processing parameters are provided in Table 2. Figure 2 shows a fast learning curve and an early plateau of the total surface mesh creation time.

**Table 2:** Surface mesh creation time

Patient	Segmentation (h)	Inspection (h)	Total time (h)	Vertices (*n*)	Triangles (*n*)
1^a^	35	14	49	397,273	795,414
2^a^	30	10	40	157,998	316,416
3^b^	13	3	16	987,349	1,795,234
4^b^	15	2	17	997,316	1,726,033
5	5	2	7	813,126	1,626,584
6	2	1	3	420,118	841,064
7	4	2	6	280,060	560,708
8	1.5	2	3.5	239,935	479,934
9^b^	9	3	12	914,674	1,678,953
10^b^	7	2	9	943,921	1,888,102
11	2	1	3	240,787	482,052
12	2	1	3	346,675	694,226
13	2	1	3	396,816	794,916
14	2	1	3	355,438	711,304
15	2	1	3	451,295	903,778
16^b^	8	3	11	897,381	1,679,743
Mean ± SD^a^	5.32 ± 4.46	1.79 ± 0.80	7.11 ± 5.08	591,778 ± 308,706	1,133,045 ± 554,784

Segmentation was done in (3D Slicer), Mesh inspection was done in Autodesk^®^ Meshmixer TM, times are estimates with 0.5 h precision

*h* hour

^a^Cases 1 and 2 not included in statistics calculation (see text)

^b^Scanning done using different modality (see text)

**Fig. 3:**
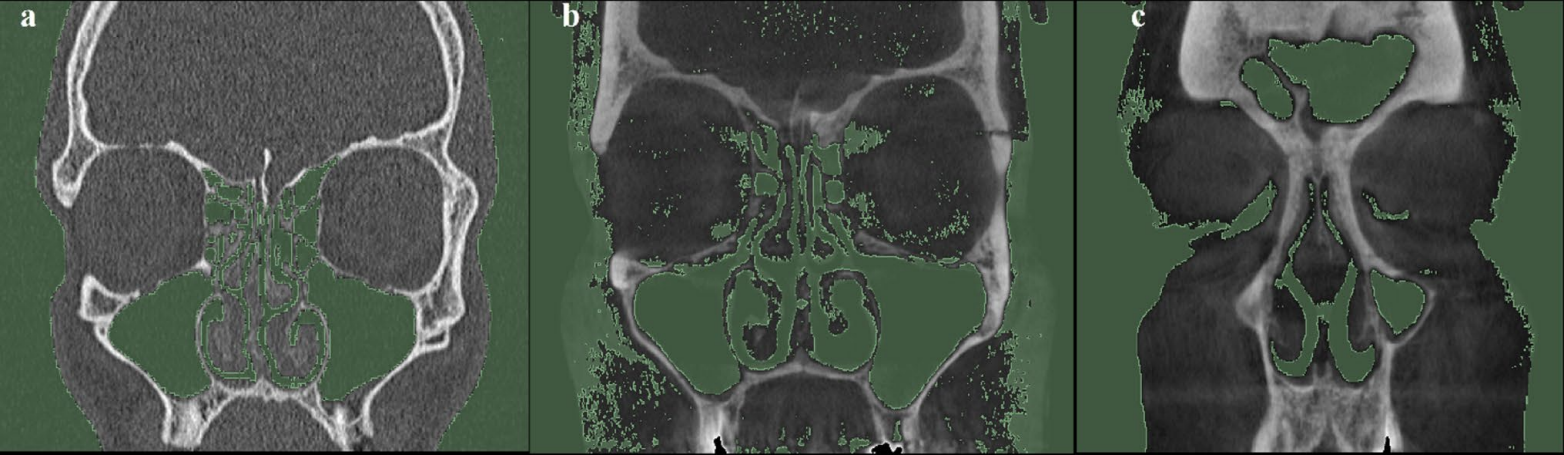
Comparison of the image during the isolation of air within the upper respiratory tract based on **a** CT (Threshold level min. − 1024 HU, max. − 300 HU) and **b** CBCT (Threshold level min. − 1024 HU, max. − 300 HU) **c** CBCT (Threshold level min. − 1024 HU, max. − 600 HU)

Figure 3 shows air isolation within the airways utilizing 3D Slicer software based on CT and CBCT techniques. It is visible that the resolution of the CT allows for the excellent marking of the airspace, see Fig. 3a. Furthermore, the CBCT study provides a lower resolution image. As a result, the change of the threshold to − 300 HU causes the selection of the soft tissue, apart from air. Also, soft tissue, nasal restrictions, and paranasal sinuses see in Fig. 3b. Further reduction of the threshold to − 600 HU leads to unselected areas of air, which does not reflect the actual condition of the respiratory tract (imitation of swelling or adhesion in the nasal cavity), see Fig. 3c.

### Computational fluid dynamics (CFD)

**Fig. 4:**
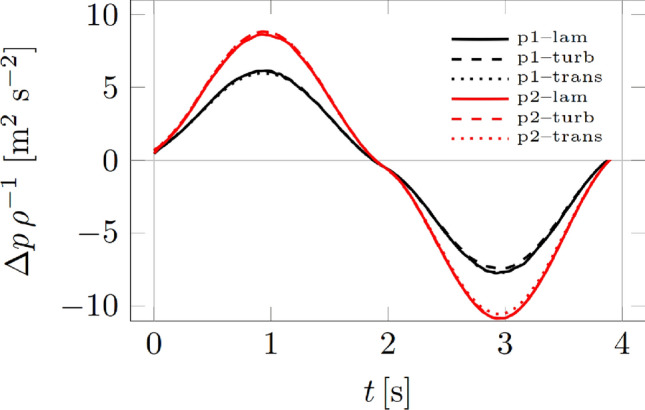
Pressure drops

**Fig. 5:**
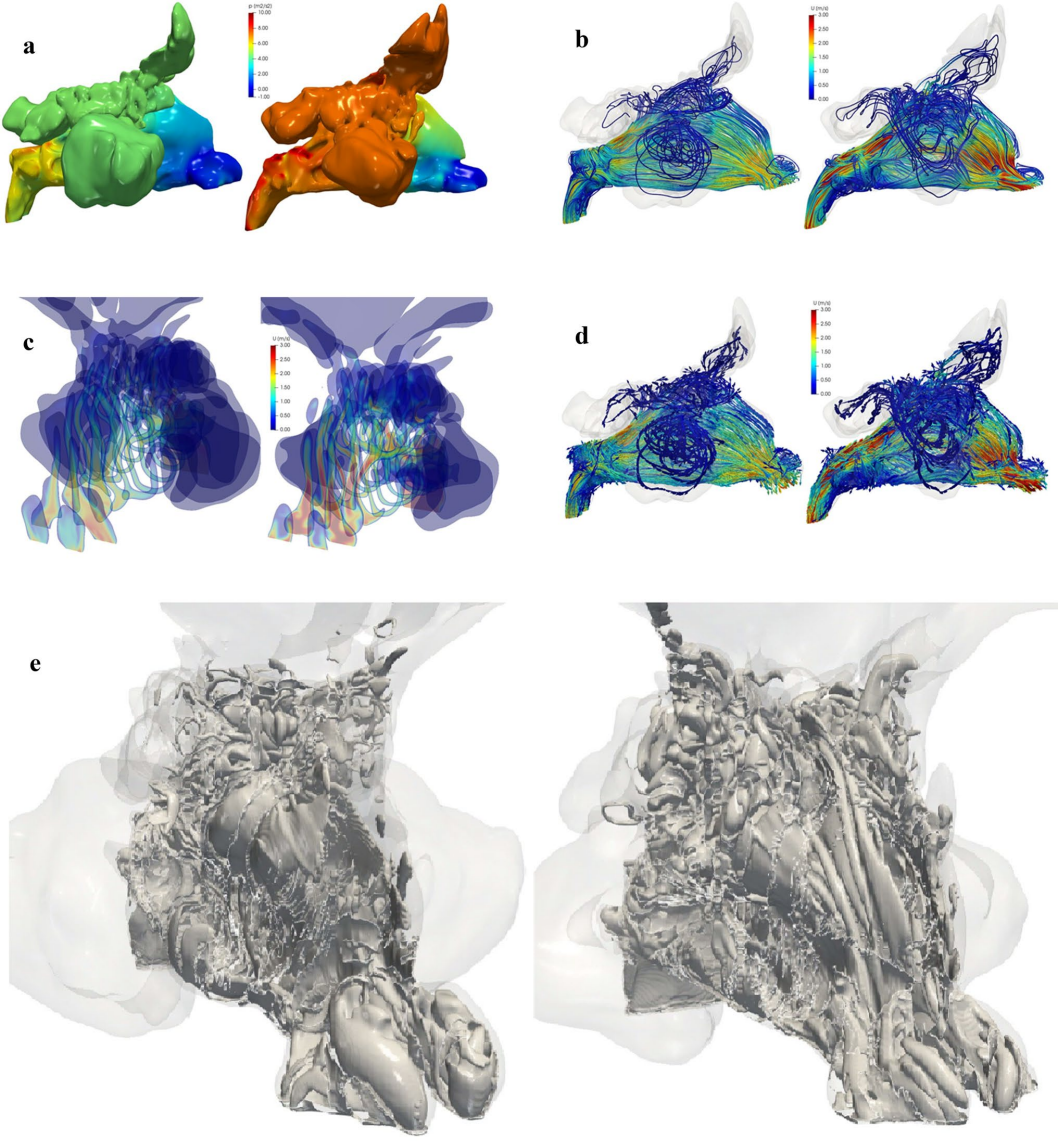
**a** Patient #1 (left) and Patient #2 (right) pressure distribution. **b** Patient #1 (left) and Patient #2 (right) trajectories. Patient #1 (left) and Patient #2 (right) velocity magnitude distributions. **d** Patient #1 (left) and Patient #2 (right) velocity vectors. **e** Patient #1 (left) and Patient #2 (right) vortex cores distribution by means of Q criterion

We present the CFD data on the example of patient 1 (normal nasal patency) and patient 2 with nasal obstruction (nasal septal deviation, concha bullosa). Figure 4 shows the pressure differences between the inlet to the flow volume (descending part of the airway to the soft palate) and the outlet (anterior nostrils). Pressures are understood here as surface averaged pressures. The calculation covers one complete inhale-exhale cycle. Figure 5a shows the pressure distributions for time *t* = 1 s, corresponding to maximum exhalation. Both figures are drawn to the same scale. The solutions were validated in two ways. First, the resistances were calculated (Table 3) and compared with the data in Kim et al. study [2]. Second, we used the *γ* *−* Re_ѳ_ transitional turbulence model, according to Langtry et al. and Menter et al., where *γ* is the intermittency and Re_ѳ_ is the transition momentum thickness Reynolds number [22, 23]. Despite the limitations of this model, in many cases it can predict the laminar-turbulent transition. The pressure drops are shown in Fig. 4 and do not differ from those obtained for laminar and turbulent flows. The results are within the ranges reported there. Table 3 also shows the mesh statistics for the two considered patients.

**Table 3:** Mesh statistics and nasal resistance (NR) [Pa s/ml]

	Patient 1	Patient 2
Nodes	12.223.696	8.269.693
Volumes	11.633.573	7.824.662
Hexahedra	11.630.016	7.821.052
Prisms	1.048	1.112
Pyramids	1.254	1.295
Tetrahedra	644	622
Polyhedra	611	581
Faces per cell	5.999	5.999
Laminar exhale	0.0792	0.1125
Laminar inhale	0.0972	0.1356
Turbulent exhale	0.0791	0.1129
Turbulent inhale	0.0972	0.1358

The mesh size corresponds to slice thickness data given in Table 1 (the minimum value is 0.25 mm). Choosing larger mesh sizes causes a loss of quality, and choosing smaller ones will not improve the quality of the scanned geometry; hence the adopted mesh size was 0.25 mm. The thickness of the boundary layers was selected so that the maximum values of y^+^ were in the range of 1–2. The height of the first layer is 0.1 mm, and the thickness ratio is 1.2. A mesh study was performed for each model separately ranging from 4.3 to 12.2 × 10^6^ and from 3.2 to 8.3 × 10^6^ nodes for patients 1 and 2, respectively. The different sizes of the computational meshes result from the different sizes of the upper respiratory tract being considered.

Figure 5b shows the trajectories that were colored with the velocity module. Additional information related to velocities is shown in Fig. 5c. They show cross-sections on which velocity distributions were plotted in the form of a velocity vector module. Figure 5d illustrates the trajectories (from Fig. 5b) with superimposed velocity vectors. The last Fig. 5e illustrates the formation of vortices in the flow by means of the Q criterion, specifically the vortex cores and vortex rings.

## Discussion

Few articles report 3-D modeling of the upper respiratory tract with further analysis of airflow by means of CFD. The results of these studies performed on small groups of patients suggest the usefulness of this approach for the assessment of airflow within the nasal cavity and paranasal sinuses [16, 19–21, 24–29]. The discussed methodology enables simulation of airflow and prediction/simulation of airflow changes in patients after nasal and paranasal sinus surgeries, for both children and adults [17, 30, 31]. Unfortunately, no randomized clinical trials on large groups of patients were published so far. Therefore it is difficult to assess the reliability of CFD results and the usefulness of the approach in medical practice.

Breathing is a dynamic phenomenon. Our methodology aims to reproduce this by applying dynamic flow values (Figs. 4, 5). The anatomical changes that happen during the breathing cycle are more difficult to the model are. The images were acquired at breath-hold, and this was assumed to represent the airway anatomy for calm breathing. It was determined anatomical changes of a nasal cavity during forceful breathing warrants a separate study requiring CT acquisition during forced inhalation and exhalation.

We analyzed the available English-language literature on the use of CFD in the analysis of airflow in the upper respiratory tract, and an attempt to describe the study protocol and the difficulties that await future researchers in this topic. The our study was based on the CT and CBCT results of 8 patients with an unchanged pathological system of the upper respiratory tract and 8 patients with an anatomical predisposition to impaired nasal breathing (nasal septal deviation, concha bullosa) has been initiated. Furthermore, the distribution of patients by age and gender have been found similar in both groups. During 3-D modeling, one consistent pattern has been observed. What is more, the CBCT result is very poorly processed and discretized by computer.

The CBCT technique is widely used in rhinology and rhinosurgery due to its wide diagnostic usefulness, sufficient image quality, and low radiological load on patients. In this present research, we noticed a significant disadvantage of CBCT: the X-ray beam collimation in CBCT leads to increased scatter radiation and degradation of image quality. CBCT scanning consists of many artifacts and decreases the contrast-to-noise ratio. Moreover, CBCT demonstrates increasing in motion artifacts because the temporal resolution of cesium iodide detectors slows data acquisition time to approximately up to 20 s [32]. However, recently published articles described 3-D modeling and CFD analysis performed on the basis of CBCT results [33, 34]. We suspect that this is due to the very low doses of radiation used in our CBCT studies, which allow for images that can be interpreted by a radiologist, but the amount of artifacts complicates 3-D airway modeling.

The 3D Slicer program has such functions as “Islands”, “Draw”, “Erase” and “Level Tracing”, which allow creating a model based on CBCT with such a high level of artifacts (Patient #10). However, it is time-consuming (Table 2, Fig. 2). Moreover, the shape of the model partially depends on the researcher (subjective feelings), and very often, the created 3-D model cannot be further discretized using CFD meshing software (Patients #3, 4, 9, and 16). Excluding the first 2 models (Patients #1 and 2), the average time to create a 3-D model of the upper respiratory tract based on CT and CBCT was 3.83 ± 1.54 h and 13 ± 3.39 h, respectively. We believe that further improvements in total time would be hard to achieve using this process while maintaining mesh quality. Possibly a dedicated, high-end, proprietary software (e.g., Materialise Mimics or Synopsys Simpleware) could offer an improvement. Nevertheless, it is possible to achieve good quality mash, useful for CFD simulations in a reasonable amount of time using open-source software.

Despite a large number of diagnostic methods and information obtained due to their implementation on the anatomical structure and respiratory capacity, it is not always possible to reliably explain the presence of ailments in the patient. Therefore, in search of answers on what is causing ailment in a patient experiencing impaired nasal breathing, we developed a workflow allowing for computer simulation of airflow through the nasal cavity and numerical analysis of the obtained data.

From Fig. 4, it can be seen that there is practically no difference between the turbulent and laminar flow for both models. The same correspondence of CFD date between laminar and turbulent models was found in other studies [31, 34]. As for the difference between individual patients, in the case of patient 2 (nasal septum deviation, concha bullosa), significantly higher pressure differences can be seen under the same boundary conditions—the same flow rate. In other words, it can be said that for Patient 2, it is necessary to create a larger pressure difference to obtain the same flow rate as for patient 1 (normal nasal patency). Furthermore, in Fig. 5, it can be seen that in the case of patient 2, the local pressure distributions reach much higher values than in the case of patient 1. The series of studies from other authors presented similar findings in patients with nasal congestion [16, 34, 35].

In Fig. 5a, b, apart from the apparent information related to the position of the fluid elements, it is seen that in the case of patient 2, the fluid elements reach locally higher velocities, which may suggest local constrictions. In Fig. 5c, as before, it can be seen that in the case of patient 2, we are dealing with higher local velocities. The CFD makes it possible to visualize not only the locations where the fluid elements travel but also their local velocity vectors, which gives additional information about directions (Fig. 5d).

The 3-D *Q*-criterion identifies vortices regarded as a spatial region where *Q* is the second invariant of velocity gradient tensor [36, 37]. More precisely, where the norm of the vorticity tensor dominates the rate of strain rate tensor norm. Invariants of velocity gradient tensors are used in turbulence modeling because they contain all the necessary information involving the rates of rotation as well as stretching and angular deformation being responsible for kinetic energy dissipation and vortex stretching [38, 39]. What is essential, the *Q*-criterion allows for direct evaluation and quantitative comparison of various flows. On the example of Patients #1 and 2, the Q criterion also illustrates the significant complication of the nasal flow (Fig. 5e). There is no information available in the literature (to our best knowledge) that describes the use of this factor as a criterion for assessing airflow through the nasal cavity.

One of the limitations of this study is that no clinical trials conducted so far on the usefulness of 3-D modeling and CFD analysis of air distribution in the nasal cavity and paranasal sinuses. So we must remember that a validation study is necessary.

It would be beneficial if future research would focus on the accuracy and precision of CDF analysis of 3-D upper airway models. Based on the above discussed limitations, it would be highly valuable to extend current studies with clinical value and cost-effectiveness study.

This study confirms the feasibility of CFD simulation of air through a nasal cavity and defines the methodology for further studies. These techniques are not expected to be applied to daily clinical routine in the nearest future, considering the need for further validation and substantial work-intensity (over 7 h of human labor per patient on average). Although the presented methodology heavily relies on technology, even more, it relies on a human operator. Intra- and interrater variability of the operator conducting segmentation, mesh repair, and finally, mesh discretization is a separate subject for further studies. It is also possible that in the future, it will be feasible to harness artificial intelligence and computer vision techniques to automate the segmentation process to eliminate the costly operator involvement and variability.

## Conclusions

In our study, we described the protocol for the preparation of a 3-D model of the nasal cavity and paranasal sinuses and highlighted several problems that the future researcher may encounter. The possibility to use freeware software along the whole workflow allows unlimited use of this method by any researcher. The training time for new user is short, even they don’t have prior experience in image segmentation and 3-D mesh editing. The disadvantages of the described research is time consuming, resource-intensive CFD calculation time and requires the necessary personnel experienced in CFD. Both CBCT and CT are useful for model building. However, CBCT may have limitations. The time-consuming creation model based on the CBCT and the quality of this model may significantly reduce the value of the study.

The *Q* criterion in CFD illustrates the considerable complication of the nasal flow and allows for direct evaluation and quantitative comparison of various flows and can be used for the qualitative assessment of airflow through the nasal cavity.

Based on our data and data from the literature, we can be concluded that the analysis of air distribution within the nose and paranasal sinuses is a perspective method of diagnostic and prognosis of the treatment. However, it requires validation of the accuracy and precision, comparison to real-life observation, assessment of clinical value, and cost-effectiveness study.
